# Bone morphogenetic protein 4 regulates immortalized chicken preadipocyte proliferation by promoting G1/S cell cycle progression

**DOI:** 10.1002/2211-5463.12640

**Published:** 2019-04-30

**Authors:** Hongyan Chen, Chang Liu, Chong Chen, Zhiyong Su, Jingting Shu, Ming Zhang, Hui Li, Bohan Cheng

**Affiliations:** ^1^ Key Laboratory of Chicken Genetics and Breeding Ministry of Agriculture and Rural Affairs Harbin China; ^2^ Key Laboratory of Animal Genetics, Breeding and Reproduction Education Department of Heilongjiang Province Harbin China; ^3^ College of Animal Science and Technology Northeast Agricultural University Harbin China; ^4^ Key Laboratory for Poultry Genetics and Breeding of Jiangsu Province Yangzhou China

**Keywords:** bone morphogenetic protein 4, chicken, preadipocyte, proliferation

## Abstract

Bone morphogenetic protein 4 (BMP4) has been reported to regulate adipose development, but its role in preadipocyte proliferation has not been explored *in vitro*. Here, we investigated the effect of BMP4 on chicken preadipocyte proliferation using immortalized chicken preadipocytes (ICP1 cells) as a cell model. We report that BMP4 expression increases during preadipocyte proliferation. Overexpression and knockdown of BMP4 promotes and inhibits preadipocyte proliferation, respectively. In addition, overexpression of BMP4 decreased the number of preadipocytes at the G0/G1 phase of the cell cycle, and increased the proportion of cells at S phase. In contrast, knockdown of BMP4 increased the number of preadipocytes at the G0/G1 phase of the cell cycle, and decreased the proportion of cells at the S and G2 phases. Furthermore, overexpression of BMP4 promoted the expression of *proliferating cell nuclear antigen* (*PCNA*), *Id2*, *cyclin E, and cyclin‐dependent kinase 2* (*CDK2*), while knockdown of BMP4 inhibited the expression of Id2, cyclin E, and CDK2. Finally, neither BMP4 overexpression nor BMP4 knockdown affected cell apoptosis. Taken together, our results suggest that BMP4 may promote proliferation of ICP1 cells by driving cell cycle transition from G1 to S phase.

**Abbreviations**
Bcl-xl: B‐cell lymphoma‐extra large
BMP4: bone morphogenetic protein 4
CCK‐8: Cell Counting Kit‐8
CDK: cyclin‐dependent kinase
CDK2: cyclin‐dependent kinase 2
CKI: CDK inhibitor
NC: negative control
PCNA: proliferating cell nuclear antigen
PI: propidium iodide
TBP: TATA‐box binding protein

The increase in adipose tissue mass is due to an increase in the number and size of adipocytes 1. Adipocyte number is regulated mainly by preadipocyte proliferation, whereas adipocyte size is regulated by preadipocyte differentiation 2. Preadipocyte proliferation is controlled by a regulatory network of multiple factors, including cytokines 3, transcription factors 4 and non‐coding RNAs 5.

Bone morphogenetic protein 4 (BMP4), a member of the transforming growth factor β superfamily originally identified based on its ability to induce ectopic bone formation 6, is now implicated in embryogenesis, organogenesis, and morphogenesis by controlling the differentiation of numerous cell types 7, 8, 9, 10. BMP4 is widely expressed in a variety of tissues 11, 12, 13, 14, 15. Accumulating evidence shows that BMP4 is a key regulator in adipose growth and development through strict control of adipocyte lineage commitment 16 and the interconversion of white fat and brown fat 17, 18. However, to the best of our knowledge, no studies have reported the role of BMP4 in preadipocyte proliferation.

Our previous study revealed that the expression of BMP4 was associated with chicken abdominal fat deposition 19, but the function of BMP4 in adipose growth and development is unknown. In the current study, we investigated the role of chicken BMP4 in preadipocyte proliferation, and the results demonstrated BMP4 promoted proliferation of immortalized chicken preadipocytes (ICP1 cells) by inducing the G1 to S phase progression.

## Materials and methods

### Cell culture and transfection

ICP1 cells were preserved in our laboratory 20.They were seeded in DMEM/F12 medium (Gibco, Grand Island, NY, USA) supplemented with 10% fetal bovine serum (Gibco) and maintained at 37 °C in a humidified, 5% CO_2_ atmosphere. After growth to 60% confluence, ICP1 cells were transfected with: (a) pCMV‐Myc‐BMP4 or pCMV‐Myc empty vector; and (b) BMP4‐siRNA or negative control (NC)‐siRNA. Three siRNAs for BMP4 and a NC were synthesized commercially (Genepharma, Shanghai, China). The siRNA sequence is shown in Table 1.

**Table 1:** The siRNA sequence used in this study.

Name	Sequence (5′–3′)
BMP4‐siRNA‐151	Sense: GCUGAUGGUCAUCCUACUATT
	Antisense: UAGUAGGAUGACCAUCAGCTT
BMP4‐siRNA‐540	Sense: GGAUCCGCUUCGUCUUCAATT
	Antisense: UUGAAGACGAAGCGGAUCCTT
BMP4‐siRNA‐872	Sense: GGCAAACACGUCAGGAUUATT
	Antisense: UAAUCCUGACGUGUUUGCCTT
NC‐siRNA	Sense: UUCUCCGAACGUGUCACGUTT
	Antisense: ACGUGACACGUUCGGAGAATT

### Cell proliferation assay

We examined cell proliferation using the Cell Counting Kit‐8 (CCK‐8) assay (Multisciences, Hangzhou, China) and EdU incorporation assay (Ribobio, Guangzhou, China). For the CCK‐8 assay, ICP1 cells were plated onto 96‐well plates at a density of 1 × 10^4^ cells/well in 100 μL of culture medium per well, and for each transfection group three independent replicates were performed. At each designated time point after transfection, 10 μL of CCK‐8 reagent was added to each well and incubated at 37 °C for 2.5 h. The absorbance of each sample at a wavelength of 450 nm was detected using a microplate reader (Molecular Devices, Sunnyvale, CA, USA). We also assessed cell proliferation using the Cell‐Light EdU DNA cell proliferation kit (Ribobio) according to the manufacturer's instructions.

### Cell cycle assay

The cell cycle was assessed using a Cell Cycle Testing Kit (Multisciences). ICP1 cells that had been cultivated in six‐well plates were harvested and centrifuged at 800 ***g*** for 5 min. The supernatant was discarded, and the cells were washed once with cold PBS. The cells were resuspended in 1 mL of kit reagent A and 10 μL of reagent B, followed by vortexing for 10 s and incubation for 30 min at room temperature, after which the cell suspension was used for flow cytometry (FACS Canto™ II; BD Biosciences, San Jose, CA, USA).

### Assessment of apoptosis

Cell apoptosis was assessed by an annexin V–FITC/propidium iodide (PI) staining assay (Multisciences). After transfection, ICP1 cells were washed with PBS two times, harvested by trypsinization, washed again with PBS, and then resuspended in 300 μL of PBS. Then cells were incubated for 15 min in the dark at room temperature in the presence of annexin V–FITC (5 μL) and PI (5 μL). Afterwards, cells were analyzed using flow cytometry, and each treatment group consisted of three replicates.

### RNA extraction and quantitative real‐time reverse transcription polymerase chain reaction

Total RNA of ICP1 cells was extracted using a TRIzol reagent kit (Invitrogen, Carlsbad, CA, USA) following the manufacturer's protocol. Total RNA was quantified using an ultraviolet spectrophotometer (Eppendorf, Hamburg, Germany) following the manufacturer's instructions. The expression levels of the genes were quantified through reverse transcription followed by real‐time polymerase chain reaction (RT‐qPCR). First strand cDNA synthesis was performed with 1 μg of total RNA (Takara, Dalian, China). The qPCR was performed using the FastStart Universal SYBR Green Master kit (Roche Molecular Systems, Pleasanton, CA, USA). A portion (1 μL) of each cDNA was amplified in a 10‐μL PCR using the ABI 7500 real‐time PCR system (Applied Biosystems, Foster City, CA, USA). The PCR conditions were one cycle at 95 °C for 10 min, followed by 40 cycles at 95 °C for 15 s and 60 °C for 1 min. Melting curves were analyzed using melting curve 1.0 software (Applied Biosystems) for each PCR to detect and eliminate possible primer–dimer artifacts. Each cDNA consisted of triplicates, and the results were analyzed using the mean of threshold cycle (*C*_t_) for each sample. Relative expression level was calculated using the $2^{-\Delta \Delta C_{t}}$ method. TATA‐box binding protein (*TBP*) was used as the reference gene 21. The primers used for qPCR were designed to span introns, and the primer sequences are shown in Table 2.

**Table 2:** Primers used for RT‐qPCR analysis.

Gene	Primer sequence (5′–3′)
*BMP4*	F: CAGATGTTTGGGCTGCGAAGG
	R: GCACGCTGCTGAGGTTGAAGAC
*Caspase‐3*	F: AAGGCTCCTGGTTTATTC
	R: CTGCCACTCTGCGATTTA
*p53*	F: CCCATCCTCACCATCCTTACA
	R: GCGCCTCATTGATCTCCTTC
*Survivin*	F: TCTACCTCGTCTCCACCCG
	R: CGCCCTTGGCTACATCTTC
*Bcl-xl*	F: GCTTTCAGCGACCTCACCT
	R: ACAATGCGTCCCACCAGTA
*Id2*	F: CCCGCAGAACAAGAAGGTCA
	R: GCTTTGCTGTCACTCGCCATTA
*Cyclin E*	F: TTTGCTATGGCTATAAGGG
	R: TGTGGTGGCGTAAGGA
*CDK2*	F: ATCAACGCCGACGGTGCCA
	R: GTGCGGAAGATACGGAAGAGC
*PCNA*	F: GTGCTGGGACCTGGGTT
	R: CGTATCCGCATTGTCTTCT
*TBP*	F: CACTTGGATGCTGGAGGT
	R: TGGAGTTGTCGGTGTAAAT

### Western blotting

For western blotting analysis, 40 μg of protein sample was mixed with 6× loading buffer (TIANGEN, Beijing, China), denatured at 95 °C for 5 min, separated by SDS/PAGE on a 12% gel and transferred to a poly(vinylidene difluoride) membrane (0.45 µm; Millipore, Bedford, MA, USA). The membrane was blocked for 2 h and then incubated overnight at 4 °C on a shaker with primary antibodies that were diluted with blocking buffer: BMP4 rabbit polyclonal IgG (USCN, PAA014Ga01, Wuhan, China). Then the membrane was incubated 1 h at room temperature on a shaker with a secondary horseradish peroxide‐conjugated antibody (Beyotime, A0208, Shanghai, China). After the membrane was washed three times with Tris‐buffered saline containing 1% Tween‐20, the target protein bands were imaged and analyzed using the Tanon‐5200 automated chemiluminescence analyzer (Tanon Science and Technology Co., Ltd, Shanghai, China).

### Statistical analysis

All experiments were repeated three times. Experimental data were analyzed using the ANOVA module of the spss statistical software version 16.0 (SPSS Inc., Chicago, IL, USA). The data were expressed as means ± SD. *P <* *0.05 represented a significant difference, and represented a highly significant difference.

## Results

### The expression of BMP4 was upregulated during proliferation of ICP1 cells

**Figure 1:**
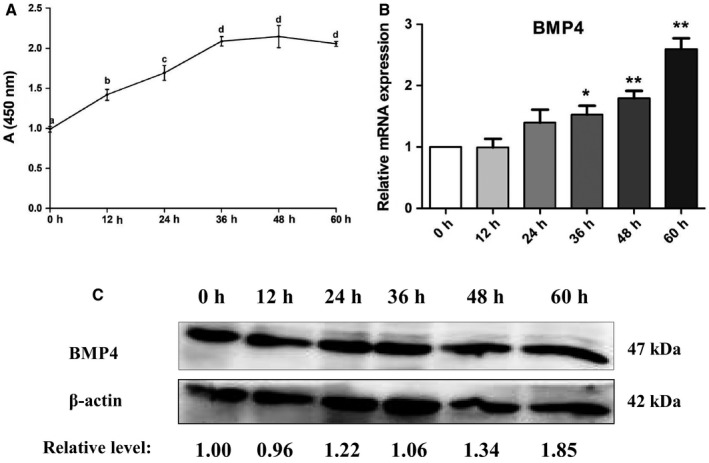
Expression of BMP4 during chicken preadipocyte proliferation. (A) Cell proliferation was measured by a CCK‐8 assay. Six hours after cell seeding was defined as 0 h for the CCK‐8 assay. (B) The mRNA expression level of *BMP4* in ICP1 cells was determined by RT‐qPCR. *TBP* was used as the internal control. (C) Western blot analyses of BMP4 proteins in ICP1 cells. Optical density of the bands was determined by image j software (Stuttgart, Germany) and normalized using an internal reference gene (β‐actin). All experiments were repeated three times. Experimental data were analyzed using the ANOVA module of the spss statistical software (version 16.0). The data were expressed as means ± SD. *P <* *0.05 represented a significant difference; and **P <* *0.01 represented a highly significant difference. ^a–d^The different lowercase letters above the line indicate significant differences for the various time points (*P *<* *0.05).

To understand whether the *BMP4 * gene was involved in chicken preadipocyte proliferation, the expression of BMP4 was detected during the proliferation of ICP1 cells. The results of a CCK‐8 assay showed that ICP1 cell number increased from 0 to 48 h, then slightly decreased at 60 h (Fig. 1A), which indicated that the cells were proliferating as normal. RT‐qPCR and western blotting showed that the expression level of BMP4 was increased during the proliferation of ICP1 cells (Fig. 1B,C).

### Effect of BMP4 on the proliferation of ICP1 cells

**Figure 2:**
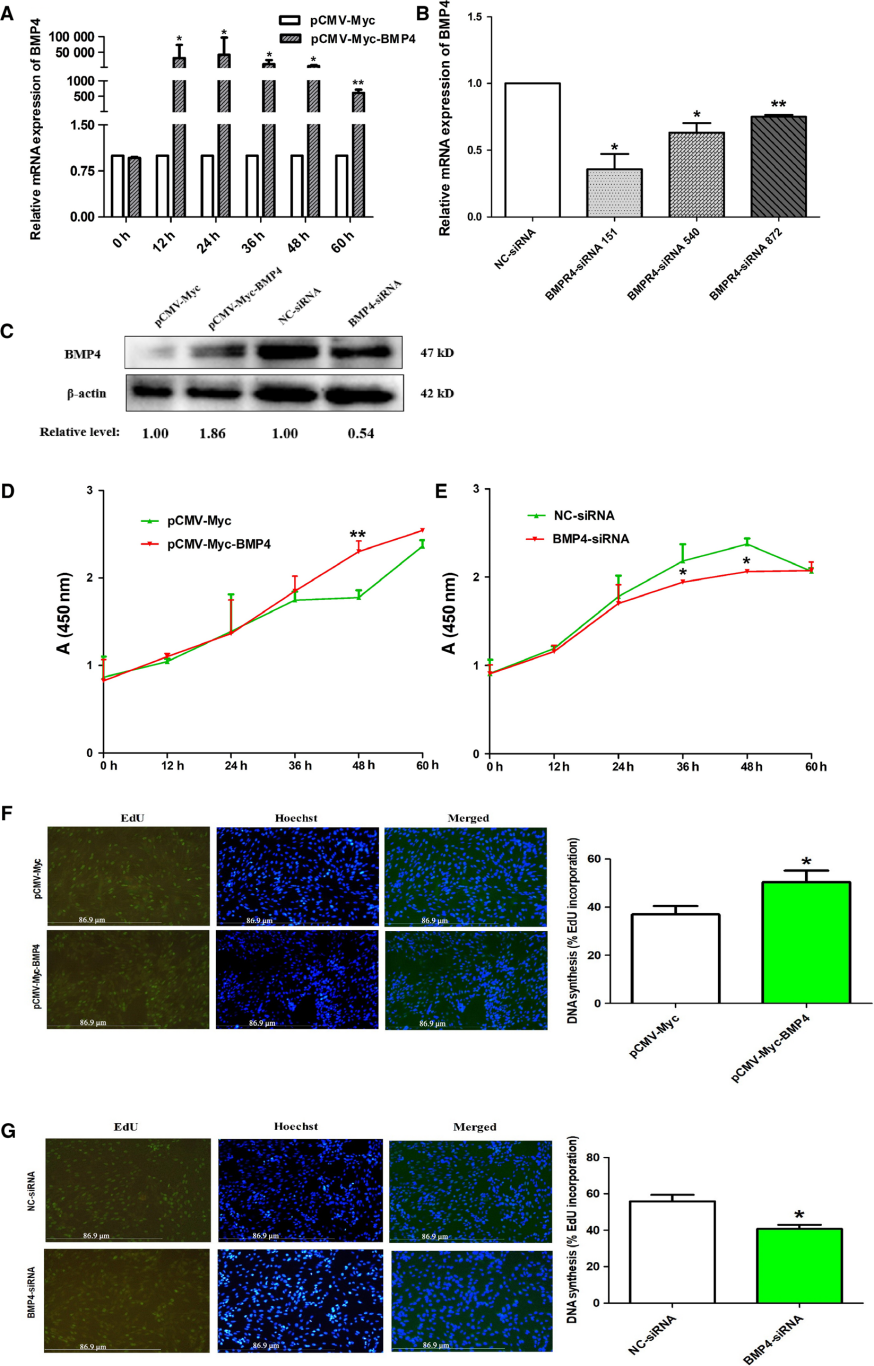
Effect of BMP4 on preadipocyte proliferation. (A) The expression of *BMP4* in ICP1 cells transfected with pCMV‐Myc‐BMP4 or pCMV‐Myc was determined by RT‐qPCR. (B) The expression of *BMP4* in ICP1 cells transfected with BMP4‐siRNA or NC‐siRNA was determined by RT‐qPCR at 36 h after transfection. (C) Western blot analyses of BMP4 proteins in ICP1 cells transfected with pCMV‐Myc‐BMP4/pCMV‐Myc, BMP4‐siRNA/NC‐siRNA. Optical density of the bands was determined by image j software and normalized using internal reference gene (β‐actin). (D, E) ICP1 cells were transfected with pCMV‐Myc‐BMP4 or pCMV‐Myc and BMP4‐siRNA or NC‐siRNA, and cell proliferation was analyzed using the CKK‐8 assay. (F, G) ICP1 cells were transfected with pCMV‐Myc‐BMP4 or pCMV‐Myc and BMP4‐siRNA or NC‐siRNA, and cell proliferation was analyzed using the EdU assay at 36 h after transfection. EdU (green) was used to detect the proliferating cells by labeling the newly synthesized DNA, and Hoechst 33342 (blue) was used to measure the background by staining total cellular DNA. The ratio EdU/Hoechst was used to evaluate newly synthesized and total DNA or the levels of cell proliferation. *TBP* was used as the internal control. ICP1 cells were photographed under a light microscope (scale bars: 86.9 µm). All experiments were repeated three times. Experimental data were analyzed using the ANOVA module of the spss statistical software (version 16.0). The data were expressed as means ± SD. *P <* *0.05 represented a significant difference; and **P <* *0.01 represented a highly significant difference.

To assess the effect of BMP4 on preadipocyte proliferation, ICP1 cells were transfected with the pCMV‐Myc‐BMP4 or pCMV‐Myc empty vector, and BMP4‐siRNA or NC‐siRNA. Firstly, the efficiencies of BMP4 overexpression and knockdown were evaluated by RT‐qPCR and western blot. The results showed that the mRNA expression of *BMP4* was dramatically increased in cells transfected with pCMV‐Myc‐BMP4 compared with those transfected with pCMV‐Myc empty vector at 12, 24, 36, 48, and 60 h after transfection (*P *<* *0.05 or *P *<* *0.01; Fig. 2A). The mRNA expression of *BMP4* was remarkably decreased in cells transfected with BMP4‐siRNA‐151, BMP4‐siRNA‐540, and BMP4‐siRNA‐872 compared with those transfected with NC‐siRNA at 36 h after transfection (*P *<* *0.05; Fig. 2B). Because the knockdown efficiency of BMP4‐siRNA‐151 was stronger than that of BMP4‐siRNA‐540 and BMP4‐siRNA‐872, BMP4‐siRNA‐151 (designated as BMP4‐siRNA) was selected for subsequent experiments. The results of the western blot also showed that BMP4 dramatically increased in cells transfected with pCMV‐Myc‐BMP4 and remarkably decreased in cells transfected with BMP4‐siRNA (Fig. 2C).

We then analyzed cell proliferation using the CCK‐8 and EdU assay. The results of the CCK‐8 assay showed that the absorbance values of the cells transfected with pCMV‐Myc‐BMP4 were significantly higher than those of cells transfected with pCMV‐Myc at 48 h after transfection (*P *<* *0.01; Fig. 2D), whereas the absorbance (450 nm) of the cells transfected with BMP4‐siRNA was significantly lower than that of the cells transfected with NC‐siRNA at 36 and 48 h after transfection (*P *<* *0.05; Fig. 2E). The results of the EdU assay showed that the DNA synthesis of the cells transfected with pCMV‐Myc‐BMP4 was significantly higher than that of cells transfected with pCMV‐Myc (*P *<* *0.05; Fig. 2F), while the DNA synthesis of the cells transfected with BMP4‐siRNA was significantly lower than that of the cells transfected with NC‐siRNA (*P *<* *0.05; Fig. 2G).

### Effect of BMP4 on the cell cycle of ICP1 cells

**Figure 3:**
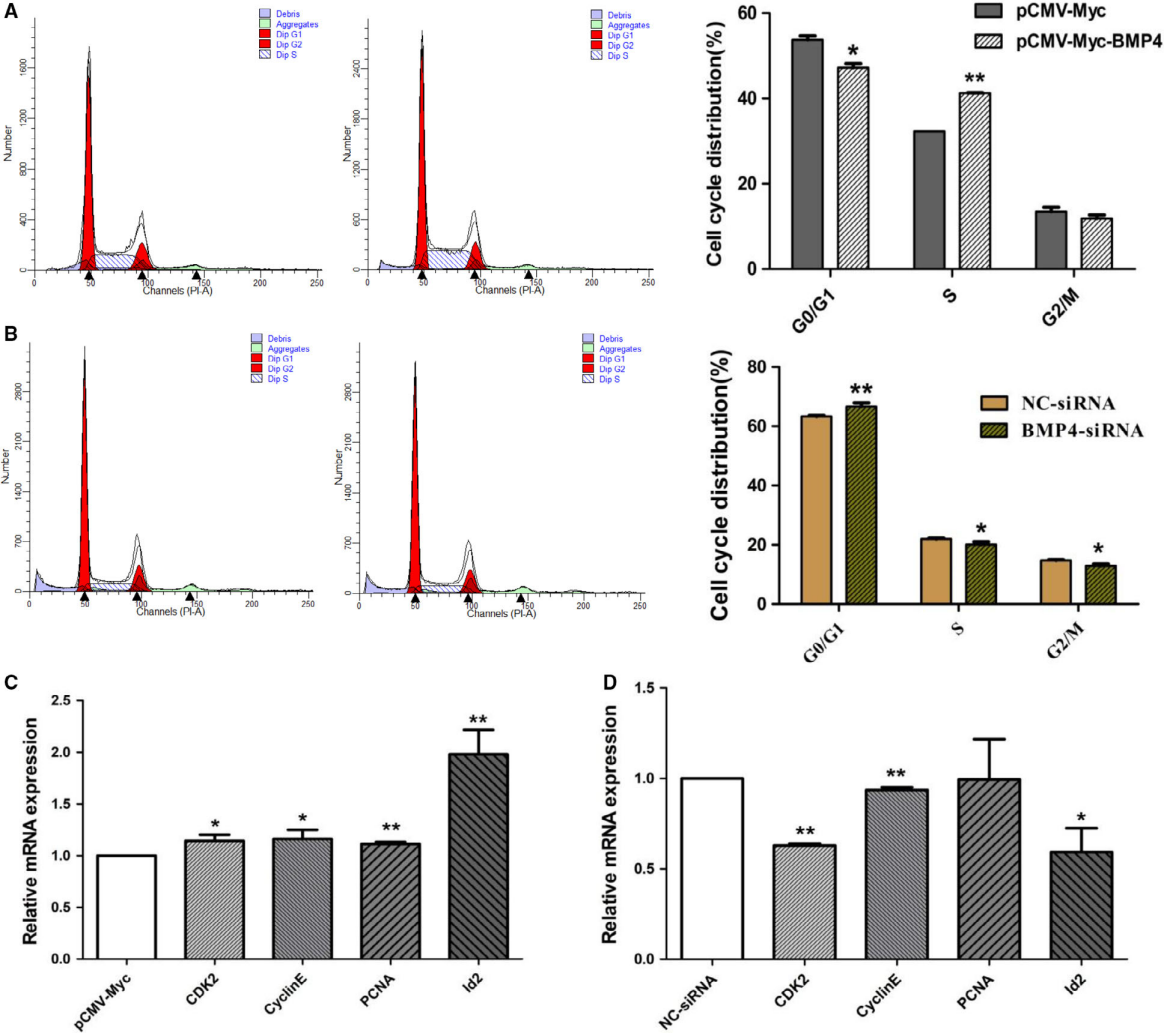
Effect of BMP4 on the cell cycle and the expression of proliferation‐related genes. (A, B) ICP1 cells were transfected with pCMV‐Myc or pCMV‐Myc‐BMP4 and NC‐siRNA or BMP4‐siRNA, and cell phases were analyzed by flow cytometry at 36 h after transfection. (C, D) mRNA expression of *CDK2*,* Cyclin E*,* PCNA*, and *Id2* was detected using RT‐qPCR at 36 h after transfection. *TBP* was used as the internal control. All experiments were repeated three times. Experimental data were analyzed using the ANOVA module of the spss statistical software (version 16.0). The data were expressed as means ± SD. *P <* *0.05 represented a significant difference; and **P <* *0.01 represented a highly significant difference.

To further investigate the role of BMP4 in preadipocyte proliferation, we assayed the cell cycle by flow cytometry. Cell cycle analysis showed that the number of cells at G0/G1 phase was distinctly decreased (*P *<* *0.05; Fig. 3A), whereas the number of cells at S phase was remarkably increased (*P *<* *0.01; Fig. 3A) among the cells transfected with pCMV‐Myc‐BMP4, compared with the cells transfected with pCMV‐Myc. In contrast, among the cells transfected with BMP4‐siRNA, the number of cells at G0/G1 phase was significantly increased (*P *<* *0.01; Fig. 3B) and the number of cells at S and G2 phase was significantly decreased (*P *<* *0.05; Fig. 3B), compared with the cells transfected with NC‐siRNA.

Furthermore, we detected the effect of BMP4 on the expression of proliferation‐related genes using RT‐qPCR. The results showed that the expression of *proliferating cell nuclear antigen* (*PCNA*), *Id2*, *cyclin‐dependent kinase 2* (*CDK2*) and *Cyclin E* was increased in the cells transfected with pCMV‐Myc‐BMP4, compared with the cells transfected with pCMV‐Myc (*P *<* *0.05 or *P *<* *0.01; Fig. 3C), whereas the expression of *Id2*,* CDK2* and *Cyclin E* was decreased in the cells transfected with BMP4‐siRNA, compared to the cells transfected with NC‐siRNA (*P *<* *0.05 or *P *<* *0.01; Fig. 3D).

### BMP4 does not influence the apoptosis of ICP1 cells

**Figure 4:**
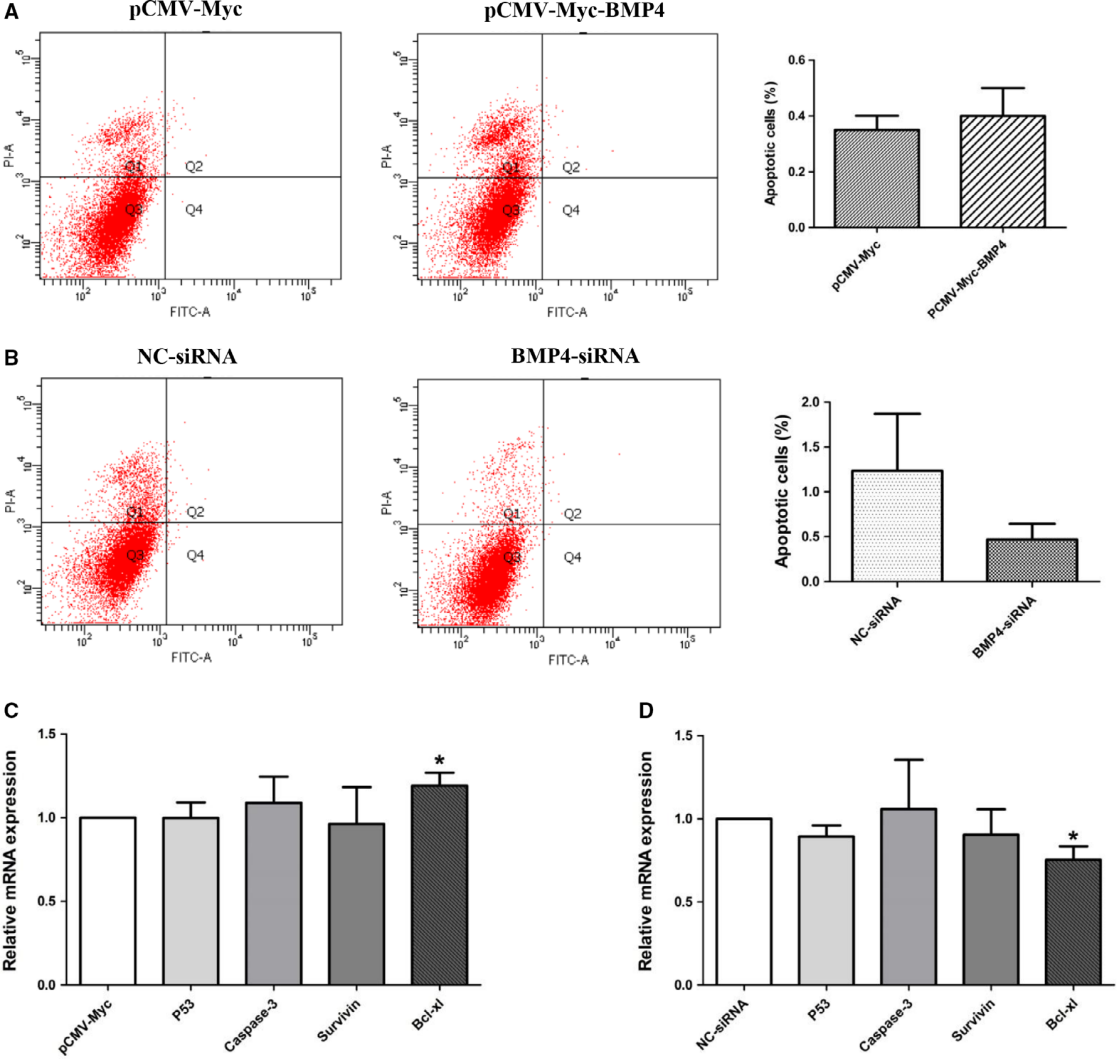
Effect of BMP4 on cell apoptosis and the expression of apoptosis‐related genes. (A, B) Cell apoptosis was determined by annexin V–FITC/PI binding followed by flow cytometry at 36 h after transfection. (C, D) mRNA expression of *p53*,* Caspase‐3*,* survivin*, and *Bcl-xl* was detected by RT‐qPCR at 36 h after transfection. *TBP* was used as the internal control. All experiments were repeated three times. Experimental data were analyzed using the ANOVA module of the spss statistical software (version 16.0). The data were expressed as means ± SD. *P <* *0.05 represented a significant difference.

It has been shown that BMP4 promoted preadipocyte proliferation (Fig. 2); thus, we investigated whether BMP4 also regulated preadipocyte apoptosis. The results of an FITC–annexin V/PI assay showed that the apoptosis of ICP1 cells was not affected by overexpression (Fig. 4A) or knockdown (Fig. 4B) of BMP4. In addition, we detected the effect of BMP4 on the expression of apoptosis‐related genes using RT‐qPCR. The results showed that the expression of *B‐cell lymphoma‐extra large* (*Bcl-xl*) was increased in cells transfected with pCMV‐Myc‐BMP4, compared with cells transfected with pCMV‐Myc (*P *<* *0.05; Fig. 4C), whereas the expression of *Bcl-xl* was decreased in the cells transfected with BMP4‐siRNA, compared with the cells transfected with NC‐siRNA (*P *<* *0.05; Fig. 4D).

## Discussion

In the current study, we demonstrated that BMP4 promoted the proliferation of ICP1 cells by inducing the G1 to S phase progression. To our knowledge, this is first report that BMP4 modulated preadipocyte proliferation.

In the current study, we found that the expression level of *BMP4* was increased during the proliferation of ICP1 cells, which indicated that BMP4 may be involved in chicken preadipocyte proliferation. Previous studies have demonstrated that BMP4 promoted the proliferation of various cell types, such as chondrocytes 22, myogenic progenitors 23, embryonic stem cells 24, and Sertoli cells 25. Consistently, our findings indicated that BMP4 overexpression enhanced preadipocyte proliferation, whereas BMP4 knockdown decreased preadipocyte proliferation. Interestingly, our finding contradicted some previous reports that showed that BMP4 inhibited proliferation of some cell types, including pancreatic α cells 26, pulmonary artery smooth muscle cells 27, and breast cancer cells 28. This discrepancy suggested that BMP4 may either promote or inhibit cell proliferation, depending on the cell type.

Cell proliferation is regulated at each phase of the cell cycle by various proteins including cyclins, cyclin‐dependent kinases (CDKs), and CDK inhibitors (CKIs) 29. Numerous studies revealed that Cyclin E and CDK2 promoted G1 to S cell cycle progression and accelerated the mitotic process 30, 31, 32. CKI could inhibit cyclins and CDKs to form complexes or inhibit complexes activity 33. In the present study, the results of flow cytometry indicated that BMP4 played a positive role in G1/S cell cycle progression, accompanied by enhanced mRNA expression of *Cyclin E* and *CDK2*. This result suggested that BMP4 promoted the proliferation of ICP1 cells by inducing G1/S progression.

Id family proteins were identified as the downstream targets of the BMP4 signaling pathway 34, 35, and were negative regulators of basic helix–loop–helix transcription factors and generally stimulated cell proliferation 33, 36. Id2 has been shown to promote G1/S cell cycle progression by releasing E2F from pRb through interaction with unphosphorylated pRb. 37. A previous study showed that BMP4 promoted proliferation and DNA synthesis of human Sertoli cells via activating Smad1/5 phosphorylation and enhancing the expression of Id2/3 25. Recently, a study revealed that BMP4 enhanced hepatocellular carcinoma proliferation by promoting cell cycle progression through upregulating the expression of Id2 38. In line with these results, the data presented in this study showed that BMP4 promoted the mRNA expression of *Id2* in ICP1 cells. This result indicated that BMP4 upregulated the expression of Id2, thereby increasing the expression of Cyclin E and CDK2, and in turn inducing the G1 to S phase transition.

Proliferation and apoptosis are the main determinants of cell survival 39. Previous studies demonstrated that p53 and caspase‐3 are proapoptotic factors 40, 41, and survivin and Bcl‐xl are anti‐apoptotic factors 42, 43. A recent study showed that BMP4 promoted neural stem/progenitor cell survival in the presence of fibroblast growth factor 2 by inhibiting apoptosis, but not by promoting proliferation 44. Furthermore, BMP4 promoted the survival of neural stem/progenitor cells by enhancing the anti‐apoptotic function of Bcl‐xl via BMP4–Smad1/5/8–Id1 signaling 44. In the present study, our findings indicated that BMP4 overexpression or knockdown did not influence the apoptosis of ICP1 cells. In addition, the expression of p53, caspase‐3, and survivin were not affected by BMP4, but the change in Bcl‐xl expression was consistent with that of BMP4 in ICP1 cells. There are several potential explanations for this observation. First, a previous study showed that p53 promotes apoptosis by repressing the expression of Bcl‐xl 45; therefore, in our study, the changes in Bcl‐xl expression may not be caused by p53, and Bcl‐xl might not play an anti‐apoptotic function in ICP1 cells. Second, Bcl‐xl inhibits apoptosis through preventing cytochrome *c* release into the cytosol and suppressing the activation of caspase‐3 46, 47, 48, but in our study, BMP4 overexpression or knockdown did not influence the expression of caspase‐3. This result further suggested that Bcl‐xl may not play an anti‐apoptotic function in ICP1 cells.

In conclusion, although further studies are required to elucidate the molecular mechanisms underlying BMP4‐mediated preadipocyte proliferation, our findings clearly identified BMP4 as a novel modulator of proliferation in ICP1 cells.

## Conflict of interest

The authors declare no conflict of interest.

## Author contributions

HC and BC conceived and designed the experiment; HC, CL, CC, ZS and BC performed experiment; JS and MZ analyzed data; HC, CL, HL and BC prepared the manuscript. All authors read and approved the final manuscript.
